# A Significant Decline of Glomerular Filtration Rate in the Majority of Long‐Term Lithium Users: Results of a Dutch Prospective 10‐Year Cohort Study

**DOI:** 10.1111/bdi.70082

**Published:** 2026-02-17

**Authors:** M. J. van der Aa, D. Zittema, J. Doornebal, E. G. T. M. Hartong, E. M. Bisseling, J. Dammers, U. M. H. Klumpers, A. P. M. Kerckhoffs, R. W. Kupka, T. Nijenhuis

**Affiliations:** ^1^ Amsterdam University Medical Center Location Vrije Universiteit Department of Psychiatry Amsterdam the Netherlands; ^2^ Amsterdam Public Health Research Institute Mental Health Amsterdam the Netherlands; ^3^ Department of Nephrology Jeroen Bosch Ziekenhuis 's‐Hertogenbosch the Netherlands; ^4^ Department of Geriatrics Jeroen Bosch Ziekenhuis 's‐Hertogenbosch the Netherlands; ^5^ Department of Nephrology Radboudumc Institute for Medical Innovations, Radboud University Medical Center Nijmegen the Netherlands; ^6^ GGZ inGeest Specialized Mental Health Care Amsterdam the Netherlands; ^7^ Amsterdam Neuroscience Mood, Anxiety, Psychosis, Sleep & Stress Amsterdam the Netherlands; ^8^ Department of Nephrology Isala Zwolle the Netherlands; ^9^ Department of Psychiatry Canisius Wilhelmina Ziekenhuis Nijmegen the Netherlands; ^10^ Jeroen Bosch Ziekenhuis, Jeroen Bosch Academy Research 's‐Hertogenbosch the Netherlands

**Keywords:** chronic kidney disease (CKD), glomerular filtration rate, lithium, maintenance treatment

## Abstract

**Introduction:**

It remains unclear to what extent long‐term lithium use leads to significant eGFR decline. This study examines the course of eGFR in a 10‐year prospective cohort of lithium users and the association with duration of lithium use, lithium serum concentration, and comedication.

**Material and Methods:**

This 10‐year prospective cohort study included patients using lithium at inclusion. Medical records were reviewed for lithium concentration, eGFR, discontinuation of lithium, and other medication use. Primary outcome was a description of eGFR decline, quantified as delta eGFR per year of follow‐up.

**Results:**

In total 196 patients were analyzed (42% male, mean age 51.1 ± 12.2, median follow‐up time 8.8 years [IQR 1.3]). Median yearly decline was 0.79 mL/min/1.73 m^2^. Of the participants, 48% had a yearly decline between 0.5 and 2.5 mL/min/1.73 m^2^, while 11% showed a decline > 2.5 mL/min/1.73 m^2^. Duration of lithium use was associated with eGFR decline. A positive association between lithium serum concentration and kidney function decline was shown, when corrected for age, sex and duration of lithium use. Comedication was not associated with eGFR decline. In the participants who discontinued lithium (20%) during follow up, there was no significant difference in eGFR before and after cessation.

**Conclusion:**

This study provides further evidence that eGFR decline occurs in most long‐term lithium users. Of this cohort, 59% of the participants had faster eGFR decline than the mean decline in the general population. Lithium exposure, quantified as mean serum lithium concentration, could be a contributing factor in this decline. eGFR trajectory was not altered by ceasing lithium.

## Practitioner Points

- In the majority of long‐term lithium users there is a significant decline in glomerular filtration rate.
- Higher mean serum lithium concentrations and longer duration of lithium therapy are associated with faster decline in glomerular filtration rate. Therefore, it seems reasonable to treat patients as short as possible with the lowest effective dose of lithium.
- In the small subset of patients who discontinued lithium during follow‐up in this cohort, no difference in eGFR decline before and after cessation was found, whether or not kidney function was the reason to stop using lithium.

## 1 Introduction

Lithium is an acute and prophylactic treatment for people with bipolar disorder. Besides its therapeutic effects, it is also associated with a number of side effects. Kidney‐associated side effects are among the most prevalent [1]. One possible kidney‐associated side effect of lithium is a decrease in kidney function (glomerular filtration rate; GFR), which may result in chronic kidney disease (CKD) and rarely in end‐stage kidney disease (ESKD), the latter estimated at a prevalence of 1.5% (95% CI 9.7‰–20.3‰) [2].

Three meta‐analyses have been published on kidney function decline in lithium‐users, all noting a significant heterogeneity between included studies. Paul et al. [3] compared lithium‐users with age‐ and sex‐matched controls in a cross‐sectional design and found that mean creatinine was slightly higher (5.7 μmol/L; 95% CI 1.7–9.9, *p* = 0.005) in patients treated with lithium. In a meta‐analysis of six studies, McKnight et al. found that the mean estimated glomerular filtration rate (eGFR) was reduced by 6.22 mL/min/1.73 m^2^ (95% BI 14.65–2.20, *p* < 0.001) in lithium users compared to non‐lithium users [1]. A more recent meta‐analysis by Schoretsanitis et al., based on 20 studies, found a prevalence of impaired kidney function (defined as an eGFR < 60 mL/min/1.73 m^2^) of 25.5% in lithium users. Compared to psychiatric patients receiving pharmacotherapy other than lithium, the odds ratio (OR) for impaired kidney function was 2.09 (95% CI 1.24–3.51, *p* = 0.005) [4].

There is debate about whether this decrease in eGFR and/or the presence of impaired kidney function is a direct result of lithium therapy. Furthermore, it is unclear whether discontinuing lithium at any time during the course of long‐term maintenance treatment results in slowing GFR decline and outweighs the benefits of continuing lithium [5, 6, 7]. Furthermore, it is not clear to what extent lithium concentration, while in the normal therapeutic range, is associated with kidney function decline in lithium users.

Since kidney function decline in long‐term lithium users is a problem often faced in daily practice by psychiatrists, questions arise about the dosage of lithium, possible discontinuation of lithium, and comedication in relation to kidney function decline. These questions require knowledge about the magnitude of kidney function decline in long‐term lithium users and knowledge about possible factors that influence this decline.

The purpose of the present study is to describe the course of kidney function in a 10‐year prospective cohort of lithium‐treated patients. The secondary aims were to analyze the relationship between the course of kidney function and total duration of lithium use, lithium serum concentration during follow‐up, and use of comedication, as well as to describe the trajectory of eGFR after discontinuation of lithium.

## 2 Material and Methods

### 2.1 Study Design and Population

The baseline characteristics of the lithium‐treated patients in this prospective cohort study at inclusion have been described in an earlier publication [8]. Briefly, people on lithium treatment in the outpatient clinics of the Canisius Wilhelmina Hospital (CWZ) Nijmegen or GGZ inGeest Mental Health Center Amsterdam were included in 2012/2013. Follow‐up period lasted until June 2023. Initially, patients of 18 years and older with a diagnosis of bipolar disorder or unipolar depression who were on lithium treatment were included.

At inclusion, information was obtained about the course of the mood disorder and the duration and interruption of lithium use. Patients were also asked about side effects experienced from lithium treatment.

The Dutch clinical practice guideline for treatment of bipolar disorder requires routine measurement of lithium concentration and eGFR minimally twice yearly [8]. Lab results and information on lithium prescription, dosage, interruptions in lithium use, and other medication used were collected from medical files. When participants discontinued treatment or were lost to follow‐up, the general practitioner or treating psychiatrist was contacted for information on eGFR and lithium treatment. Subjects with less than one‐year follow‐up were excluded.

Kidney function was based on eGFR and calculated from serum creatinine values using the Chronic Kidney Disease Epidemiology Collaboration (CKD‐EPI) equation [9]. To determine the mean eGFR trajectory, short‐term fluctuations in eGFR, such as a temporary increase during pregnancy, were excluded. Baseline eGFR was defined as the mean eGFR in the first 90 days after the initial obtained eGFR, including this value. The same principle was applied to the follow‐up eGFR, which was defined as the mean of any eGFR measured in the last 90 days before the last eGFR (including the last eGFR). All eGFR trajectories were independently evaluated by two experienced nephrologists to review the trajectories and identify any highly unexpected measurements, interpreted as outliers. CKD stages were defined conform the KDIGO guidelines [10].

The study protocol was approved by the Central Ethics Committee on Research involving human subjects Oost Nederland and the study was conducted according to the principles of the Declaration of Helsinki. Written informed consent for participation and inspection of the medical file was obtained from all participants.

### 2.2 Statistical Analysis

Data management and analysis were performed using SPSS 27.0 [11]. Subjects were divided arbitrarily into three groups based on yearly eGFR decline, in accordance with normal kidney function development versus pathological decline: less than 0.5 mL/min/1.73 m^2^, 0.5 to 2.5 mL/min/1.73 m^2^ and more than 2.5 mL/min/1.73 m^2^.

eGFR at inclusion, at cessation of lithium and at end of follow‐up were compared using a repeated measures ANOVA. eGFR decline per year was calculated during the full follow‐up time before and after cessation of lithium use and compared using a Wilcoxon Signed Rank test. Yearly eGFR decline was compared between patients ceasing lithium versus continuers using a Mann–Whitney *U* test. Change in eGFR decline per year after ceasing lithium was compared between participants who ceased using lithium because of eGFR decline (as stated in their medical file) and participants who ceased using lithium due to other reasons using an unpaired *t*‐test. A one‐way ANOVA was conducted to establish the relation between eGFR decline and age at follow‐up. The relation between lithium concentration and kidney function decline was analyzed through a mixed‐effects model. Sex, duration of lithium use at baseline and age at inclusion were included as covariates. In addition, we included random intercepts for unique patient codes in order to compare the different individual eGFR trajectories between individuals using this model. Furthermore, the interaction between time and lithium concentration was included. The possible association between eGFR decline (as divided in three categories: less than 0.5 mL/min/1.73 m^2^, 0.5 to 2.5 mL/min/1.73 m^2^ and more than 2.5 mL/min/1.73 m^2^) and duration of lithium use was tested using the Kruskal–Wallis test. Using a mixed model analysis, the relationship between comedication known to be putatively nephrotoxic or influence eGFR (anticonvulsants, thiazide diuretics, furosemide, Non Steroid Anti Inflammatory Drug's [NSAID's] and proton pump inhibitors [PPI]) and eGFR decline was analyzed and corrected for sex and age at inclusion, since these factors are known to influence eGFR decline [12].

## 3 Results

In this cohort, 201 subjects (42.3% male) were initially included in 2012/2013. After evaluation of the follow‐up duration in 2023, five participants were excluded due to insufficient follow‐up time (2.5%), resulting in a cohort of 196 subjects. The 15 subjects (7.7%) that died during follow‐up were included in the analysis up to the moment of decease. Demographic and clinical characteristics are displayed in Table 1.

**TABLE 1:** Demographic and clinical characteristics at baseline and follow‐up.

Male sex (*n*, percentage)	83 (42%)
Drop out due to decease (*n*, percentage)	15 (7.7%)
Baseline age (mean age in years, SD)	51.1 (12.2)
Age at follow‐up (mean age in years, SD)	58.9 (12.4)
Baseline eGFR (mean eGFR in ml/min/1.73 m^2^, SD)	81.5 (17.2)
eGFR at follow‐up (mean eGFR in ml/min/1.73 m^2^, SD)	74.4 (20.1)
Baseline duration of lithium use (median years, IQR)	8.0 (4–14)
Follow‐up duration (median years, IQR)	8.8 (7.8–9.1)
Mean lithium concentration (mmol/L, SD)	0.70 (0.2)
Number of lithium measurements per patient (mean, SD)	25.2 (13.8)
Comedication	
Anticonvulsants (*n*, percentage)	42 (21.4%)
Thiazide diuretics (*n*, percentage)	13 (6.6%)
Furosemide (*n*, percentage)	2 (1.0%)
NSAID's (*n*, percentage)	5 (2.6%)
PPI (*n*, percentage)	23 (11.2%)

Abbreviations: eGFR, estimated glomerular filtration rate; IQR, interquartal range; NSAID, Non Steroid Anti Inflammatory Drug; PPI, Proton Pump Inhibitor; SD, standard deviation.

### 3.1 eGFR Trajectory

A significant difference between eGFR at inclusion (mean 81.5 mL/min/1.73 m^2^ [±16.9]) and at end of follow‐up (mean 74.4 mL/min/1.73 m^2^ [±20.5]) was found (*p* < 0.001) for the total cohort. Median follow‐up duration was 8.7 years (IQR 1.3).

The trajectories of the eGFR were linear in most patients. However, eGFR measurements during clinical hospital admissions and pregnancies were often non‐linear, and those periods were therefore excluded in all patients. The median change of eGFR per year was 0.79 mL/min/1.73 m^2^ (IQR 1.93).

As shown in Table 2, 11% of the participants had > 2.5 mL/min/1.73 m^2^ eGFR decline per year. There was no significant difference in mean age between the different categories of eGFR decline (Table 2).

**TABLE 2:** Results of eGFR decline per year divided in categories.

eGFR decline	Number of subjects, percentage	Median duration of lithium use in years, IQR^a^	Mean age in years at follow‐up, SD (NS)
Total group	196 (100%)	16 (12–21)	58.9 (12.4)
< 0.5 mL/min/1.73 m^2^ per year	81 (41%)	14.0 (10–18)	56.5 (13.0)
0.5–2.5 mL/min/1.73 m^2^ per year	94 (48%)	16.3 (13–23)	60.4 (11.8)
> 2.5 mL/min/1.73 m^2^ per year	21 (11%)	22.1 (14–28.3)	61.1 (11.5)

Abbreviation: NS, not significant.

^a^ Groups are significantly different using the Kruskal–Wallis test (*p* < 0.001).

At inclusion, 89.7% of the participants had a CKD stage G1 or G2 (eGFR 60–89 mL/min/1.73 m^2^), 8.7% had stage G3a (eGFR 45–59 mL/min/1.73 m^2^), 1.5% had G3b (eGFR 30–44 mL/min/1.73 m^2^), and none had stage G4 (eGFR 15–29 mL/min/1.73 m^2^) or G5 (eGFR < 15 mL/min/1.73 m^2^). At the end of follow‐up, 77% of the participants had CKD stage G1 or G2, 14.3% had stage G3a, 6.6% had G3b, and 2% had stage G4.

### 3.2 eGFR Decline and Lithium Concentration

Mixed model analysis showed a significant association between lithium concentrations and eGFR decline (*b* = −3.7, 95% CI −5.7 to −1.8) when controlled for age, sex and duration of lithium therapy at baseline.

### 3.3 Duration of Lithium Use

The median duration of lithium use was 16.0 years (IQR 11). Median duration of lithium use was significantly associated with higher yearly reduction in eGFR (*p* < 0.001).

### 3.4 Comedication

No significant association between the use of high‐risk comedication at baseline (anticonvulsants, thiazide diuretics, furosemide, NSAID's and proton pump inhibitors) and yearly eGFR decline when corrected for sex and age was found (*b* = −3.7, 95% CI −8.6 to 1.2).

### 3.5 Discontinuation of Lithium

**FIGURE 1:**
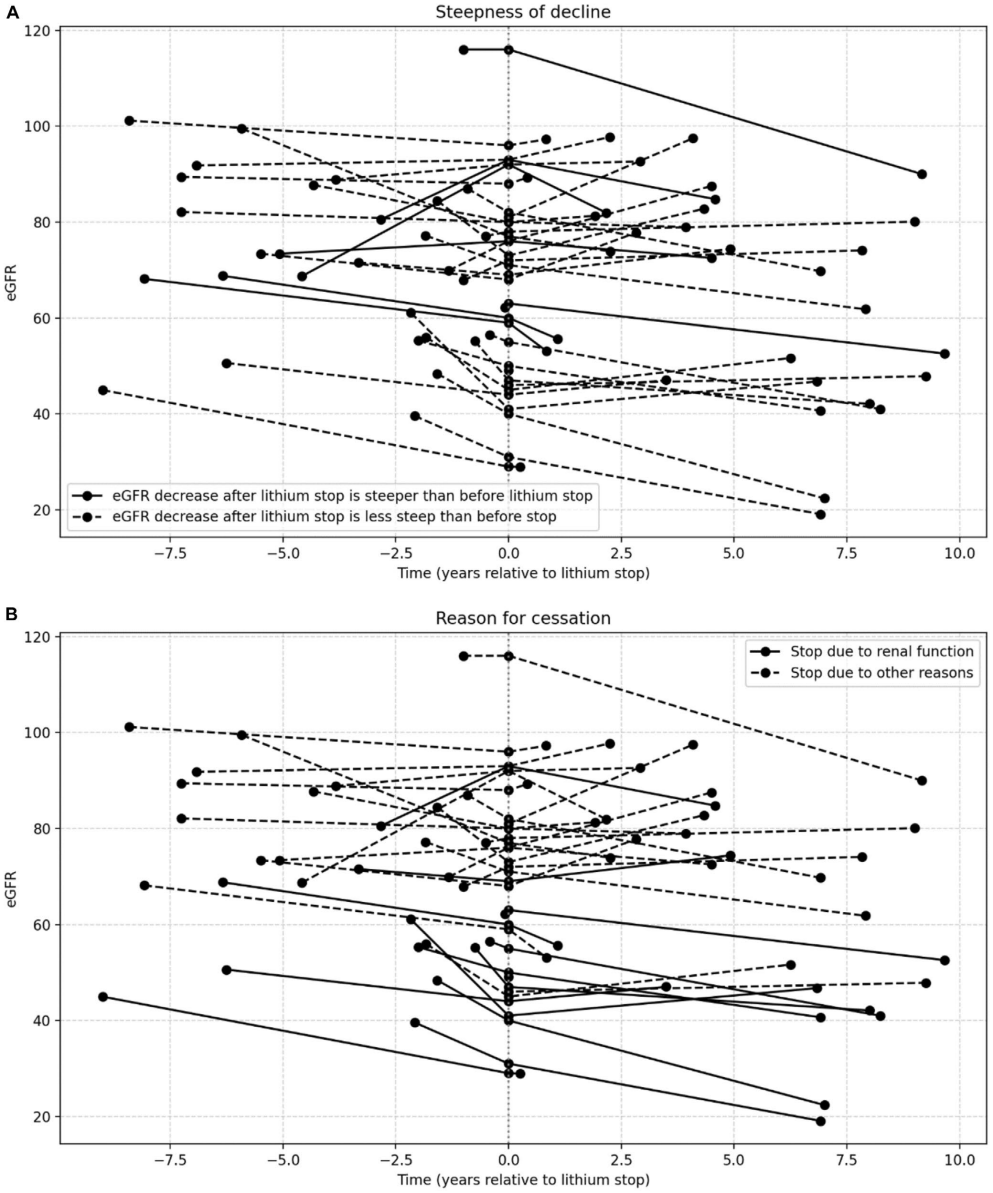
Individual eGFR trajectory (*n* = 33) before and after lithium cessation. T = 0 is stop moment of lithium. “A” highlights the participants who have a steeper eGFR decrease after stopping lithium. “B” compares participants who discontinued lithium due to a reduced renal function to participants who stopped for other reasons.

Of the 196 subjects, 39 (20.4%) discontinued lithium during follow‐up. Discontinuation was reported to be decided upon because of presumed kidney function decline or suggested nephrotoxicity (*n* = 11, 5.6%), complaints of polyuria (*n* = 2, 1.0%) or psychiatric remission (*n* = 5, 2.6%), while 18 participants had other reasons for stopping lithium (e.g., various other side effects). In three participants the reason for stopping lithium was not documented. There was no significant difference in yearly eGFR decline between participants who discontinued lithium (before discontinuation of lithium) and those who continued (median yearly decline of 1.1 vs. 0.8 mL/min/1.73 m^2^, *p* = 0.149). In 33 of these 39 participants follow‐up information after stopping lithium was available. In people who discontinued lithium, mean eGFR at inclusion was 74.4 mL/min/1.73 m^2^ (SD 17.8), at the moment of stopping lithium 70.2 mL/min/1.73 m^2^ (SD 3.5) and at final follow up 69.7 mL/min/1.73 m^2^ (SD 23.1). Median follow‐up duration after stopping lithium was 3.7 years (IQR 6.25) (Figure 1). Median yearly decline before stopping lithium was 1.3 mL/min/1.73 m^2^ (IQR 4.8) and median decline after lithium discontinuation was 0.27 mL/min/1.73 m^2^ (IQR 2.66) per year. Although numerically there was a lower rate of decline after stopping lithium, this did not differ significantly. Four participants who stopped lithium had more than 2.5 mL/min/1.73 m^2^ per year decline and 11 showed between 0.5 and 2.5 mL/min/1.73 m^2^ per year decline after discontinuation.

In the 11 participants who had kidney function as the main reason for discontinuing lithium, the median yearly decline in eGFR from inclusion to stopping lithium was 3.1 mL/min/1.73 m^2^ (IQR 5.2) and from stopping lithium to end of follow‐up 1.0 mL/min/1.73 m^2^ (IQR 2.8) (Figure 1), again a non‐significant difference. Furthermore, the decline before lithium discontinuation did not differ in the participants who had kidney function as the main reason for discontinuing lithium compared to all other participants (*p* = 0.063). In the 22 participants who stopped using lithium for other reasons, the median yearly decline in eGFR from inclusion to stopping lithium was 0.45 mL/min/1.73 m^2^ (IQR 5.2) and from stopping lithium to end of follow‐up −0.2 mL/min/1.73 m^2^ (IQR 2.8). There was no significant difference in yearly eGFR decline after discontinuing lithium between participants who stopped lithium because of kidney function decline and participants who stopped lithium due to other reasons.

## 4 Discussion

In this study, 59% of long‐term lithium users developed a decline in eGFR that was more than 0.5 mL/min/1.73 m^2^ per year, 48% of the participants had a decrease in eGFR between 0.5 and 2.5 mL/min/1.73 m^2^ per year, and 11% showed an eGFR decrease of more than 2.5 mL/min/1.73 m^2^ per year. The observations show a median decrease in eGFR of 0.79 mL/min/1.73 m^2^ per year over a medium follow‐up period of 8.7 years. When comparing this to the general population, the Nijmegen Biomedical Study estimated a yearly decrease of 0.4 mL/min/1.73 m^2^ per year in the general (Dutch) population [13]. Thus, the mean decline in our cohort is more than to be expected. This is in accordance with earlier research, suggesting an accelerated eGFR decline associated with long‐term lithium use [1].

After a median duration lithium use of 16 years, the incidence of CKD stage G4 or higher (CKD4+, an eGFR < 30 mL/min/1.73 m^2^) was small (2%, *n* = 4). In other research on CKD in long‐time lithium users, a 10‐year cumulative risk on CKD4+ in people who started lithium between ages 18 and 54 between 0% and 0.7% was found. In people who started lithium at a more advanced age, the lifetime risk of CKD4+ was higher, ranging from 13.9% to 18.6%. Since this study included people who started lithium at a wide range of ages (ranging from 16 to 77 years old), the incidence of 2% is in accordance with these results [14].

It has been argued that the decline in eGFR is not directly related to lithium treatment itself, but due to comorbidities and the use of other nephrotoxic drugs [15]. In this population we could not find any evidence that the nephrotoxic drugs that were analyzed (NSAID's, PPI's, anticonvulsants, furosemide and thiazide diuretics) could be a confounding variable in the relationship between lithium use and kidney function decline. However, the list of analyzed drugs was limited and did not include any details about the length of comedication use. Comorbidities were not included in this analysis.

In this study we found that there is an association between lithium concentrations and duration of lithium use and decline in eGFR. This is in accordance with earlier research which showed that longer duration of lithium use, advanced age and kidney function impairment at baseline are positively associated with kidney function decline in lithium users [4, 15, 16, 17, 18, 19]. Furthermore, lithium dosage (based on filed prescriptions) proved a factor of influence in this relationship [20]. Since drug prescriptions are not a reliable source of information on actual drug use, other research has focused more on the association of kidney function decline with lithium intoxications and lithium concentration.

Lithium intoxications, which are often defined variably, seem associated with kidney function decline in lithium users [15, 21, 22]. When examining the literature on serum lithium concentration (not only defined as intoxications), diverging results are found. Some studies did find an association between lithium concentrations and a reduced kidney function or kidney function decline [17, 23, 24, 25]. Other studies failed to find any relationship between lithium concentration and kidney function [21, 26, 27]. When examining these studies, lithium concentration is defined in various ways. Often, a mean or median serum lithium concentration is calculated or a single lithium measurement is used. In this study, we included all individual measurements of lithium concentration during follow‐up period in a mixed model analysis, which showed a significant relationship between higher serum lithium concentration and eGFR decline. This substantiates a direct causal relation between lithium treatment and kidney function decline. In clinical practice, a tendency towards prescribing lithium at the lowest effective serum concentration may be an explanation for the lower decline in kidney function found in this study in comparison to earlier studies.

In a Delphi study design, optimal prophylactic serum lithium concentration was found to be 0.60–0.80 mmol/L in general, to be lowered to 0.40–0.60 mmol/L in people with good response but with severe side effects (such as kidney function decline) [28]. Our results confirm the proposition of a relationship between lithium serum concentration and the decline in kidney function. Therefore, it is advisable to pursue the lowest lithium concentration necessary to achieve optimal clinical outcomes, particularly mood stability.

Different guidelines contain varying recommendations on how often kidney function should be measured, ranging from three to 6 months [29]. Psychiatrists were asked how frequently they assessed kidney function in patients undergoing maintenance lithium therapy [30]. None reported not to measure kidney function; 21% measured it once per year, while 51% assessed kidney function two to three times per year. Based on our findings of kidney function decline in lithium users being a slow and gradual process, it is important to establish its course using regular measurements. Interpretation of these measurements should not only focus on sudden decline (e.g., due to dehydration or lithium intoxication) but also on gradual and constant decline over a long period of time. Visualizing the kidney function trajectory over years (e.g., in a graph) could be helpful.

Irrespective of the reason for discontinuation of lithium, participants who discontinued lithium during follow‐up showed no significant difference in the mean eGFR decline after discontinuing lithium compared to before discontinuing lithium. Earlier studies have found diverging results when examining kidney function after withdrawal of lithium. These studies have a similar small number of participants (27 and 43) [6, 7]. In our study, the mean eGFR decline after stopping lithium is numerically lower without reaching statistical significance, which could be a result of low numbers of subjects ceasing lithium, let alone ceasing lithium because of actually documented kidney function decline. Some studies suggest a point of no return for eGFR between 30 and 40 mL/min/1.73 m^2^, below which no amelioration of eGFR decline after stopping lithium was found [6, 31]. We were not able to confirm this in our cohort, since no specific point, below which no participants had improvement of the decline in eGFR after stopping lithium, was identified.

Taken together, we demonstrated that in the majority of patients with mood disorders in our cohort who use lithium, the decline of kidney function is faster than expected in the general population based on available data from the general population. This decline is related to lithium concentration. For clinical practice and considering variations in eGFR that occur, we recommend measuring lithium concentration regularly (according to guidelines) as well as kidney function at individualized intervals (e.g., between 3 and 6 months). Given the often relatively slow decline in kidney function due to lithium toxicity over time, we recommend making yearly assessments of kidney function decline part of clinical practice. When kidney function decline exceeds 2.5 mL/min/year, referral to a kidney specialist should be considered in order to rule out other causes of kidney function decline. Based on our findings, we cannot make any recommendations on whether or not lithium therapy should be ceased in people experiencing kidney function decline.

We previously proposed a decline in eGFR of 30% or more in at least 1 year as a possible surrogate marker for End stage kidney disease (ESKD) [32]. However, since there is a lack of research on such surrogate markers in lithium treatment, we sought studies in populations that resemble the lithium users to identify this possible surrogate marker. This, or any other marker, still needs confirmation in prospective studies in lithium users before being applicable in clinical practice to guide treatment. Since we did not see many people reaching end‐stage kidney disease, we cannot confirm such surrogate marker in the cohort in this current paper. Further studies are needed to establish whether the long‐term kidney function decline can be attributed solely to lithium use. Discontinuation of lithium in our cohort numerically reduced renal function decline (1.3 to 0.27 mL/min/1.73 m^2^/year), without reaching statistical significance, possibly due to low number of patients discontinuing lithium in our cohort. An additional explanation may be that the follow‐up period after lithium discontinuation was insufficient to detect longer‐term differences. Future research should focus on the two most important clinical questions that arise when prescribing lithium: what is the (lifetime) risk of clinically relevant kidney function decline use at start of therapy, and what is clinically relevant decline in this population, as well as if and when should the decision be made to discontinue lithium in case of kidney function decline. In order to investigate this, a larger cohort should be followed over more than 10 years and should be compared to an adequate control group. Alternatively, biomarkers should be sought that function as surrogate measures of (future) kidney function decline to be able to preclude the need for decade‐long clinical studies.

The strength of our observational study is the prospective nature, the relatively long follow‐up time, and the fact that the participants have been on long‐term lithium treatment. Due to the frequent regular protocolized kidney function monitoring of patients using lithium of a minimum of two times per year, a comprehensive overview of the trajectories of the kidney function could be constructed, as exemplified by the 25.2 measurements of lithium concentration over a median follow up duration of 8.8 years. A high number of serum lithium concentration measurements, with a total of 1834 measurements included, led to a reliable estimate of the serum lithium concentration over the follow‐up period. However, several limitations to this study need to be addressed. When measuring serum lithium concentration, timing is important. Lithium measurements should always take place 12 h after lithium intake [28]. This is common practice in the Netherlands. However, since this was a pragmatic, clinical study and not a research design, this was not in the protocol and could not be verified. The observational origin makes it impossible to prove causal relationships. A second limitation is that information on co‐medication was only collected at baseline and further extracted from medical records, which limits the reliability of obtaining a comprehensive overview of medication use and treatment duration. Based on this analysis, this does not exclude the possibility that there could still be an association between total or peak exposure to co‐medication. Another limitation is the lack of a (diseased) control group. Ideally, such a control group would have been composed of people with bipolar disorder using long‐term (non‐nephrotoxic) psychotropic medications other than lithium.

## 5 Conclusion

In conclusion, in this cohort study we found that most long‐term lithium users experience a significant decrease in eGFR. Higher serum lithium concentrations and longer therapy duration are associated with a higher decline in eGFR. In 11% of our cohort, an eGFR decline of > 2.5 mL/min/year was demonstrated. Only a small subset of patients discontinued lithium, and in this group the eGFR decline was not statistically different before or after discontinuation, whether or not kidney function was the reason to stop, possibly due to limited power and short follow‐up.

## Funding

This work was supported by the Stichting Jos Schaap Feering.

## Conflicts of Interest

The authors declare no conflicts of interest.

## Data Availability

Research data are not shared.
